# Supplementary material for: Causes and consequences of sex-chromosome turnovers in Diptera
Source: Evol Lett. 2026 Mar 12;10(3):253–68. doi: 10.1093/evlett/qrag003 (PMC13231274; doi:10.1093/evlett/qrag003)
Supplement: qrag003_Supplemental_Files [file qrag003_supplemental_files.zip › Supplementary_Datasets.zip!/Supplementary_Datasets/Dataset_2_Graphical_representation_outgroups/Drosophilidae_Drosophila_triauraria_chromosomes.pdf]

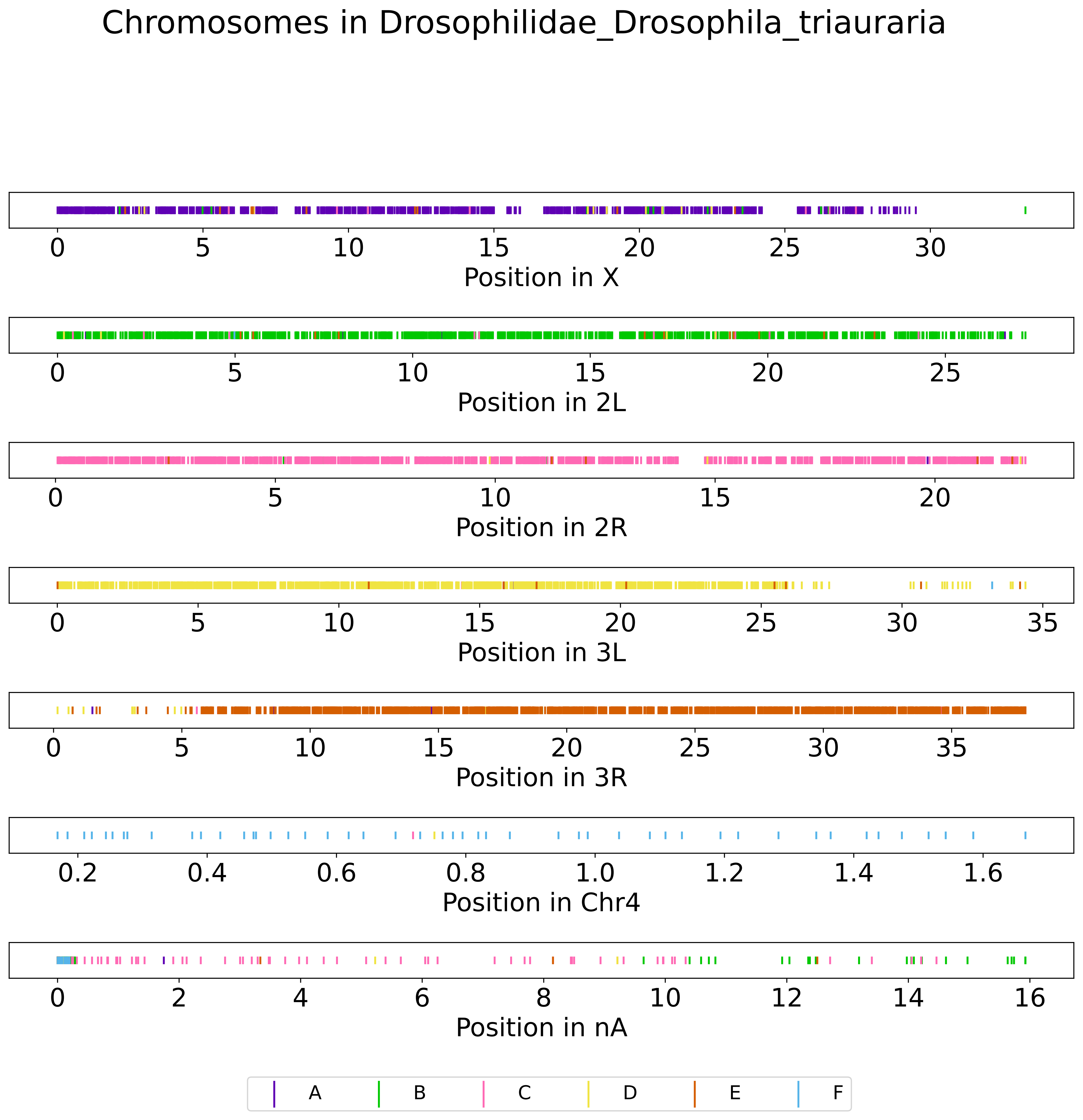
Chromosomes in Drosophilidae_Drosophila_triauraria
0 5 10 15 20 25 30
Position in X
0 5 10 15 20 25
Position in 2L
0 5 10 15 20
Position in 2R
0 5 10 15 20 25 30 35
Position in 3L
0 5 10 15 20 25 30 35
Position in 3R
0.2 0.4 0.6 0.8 1.0 1.2 1.4 1.6
Position in Chr4
0 2 4 6 8 10 12 14 16
Position in nA
A
B
C
D
E
F
